# Supplementary material for: Sesame as an Alternative Host Plant to Establish and Retain Predatory Mirids in Open-Field Tomatoes
Source: Plants (Basel). 2022 Oct 20;11(20):2779. doi: 10.3390/plants11202779 (PMC9612361; doi:10.3390/plants11202779)

A: Cage Study Treatments

1. *B. tabaci* + *N. tenuis* 4 plants	2. *B. tabaci* + Insecticide 4 plants	3. *B. tabaci* + Untreated Tomato 4 plants

B: *B. tabaci* (Eggs + Nymphs)

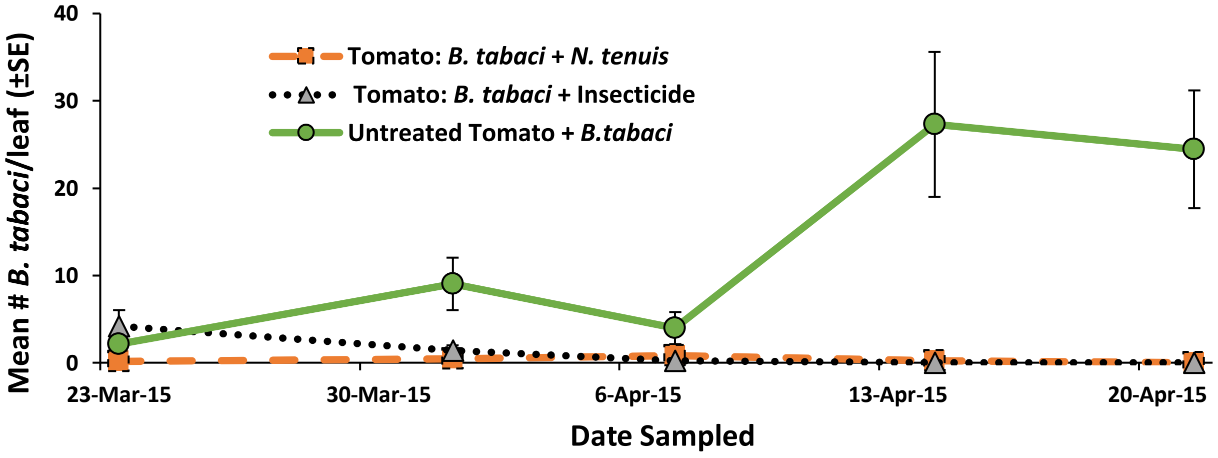

C: Cage Study *N. tenuis* (Adults+ Nymphs) and Damage (Necrotic Rings)

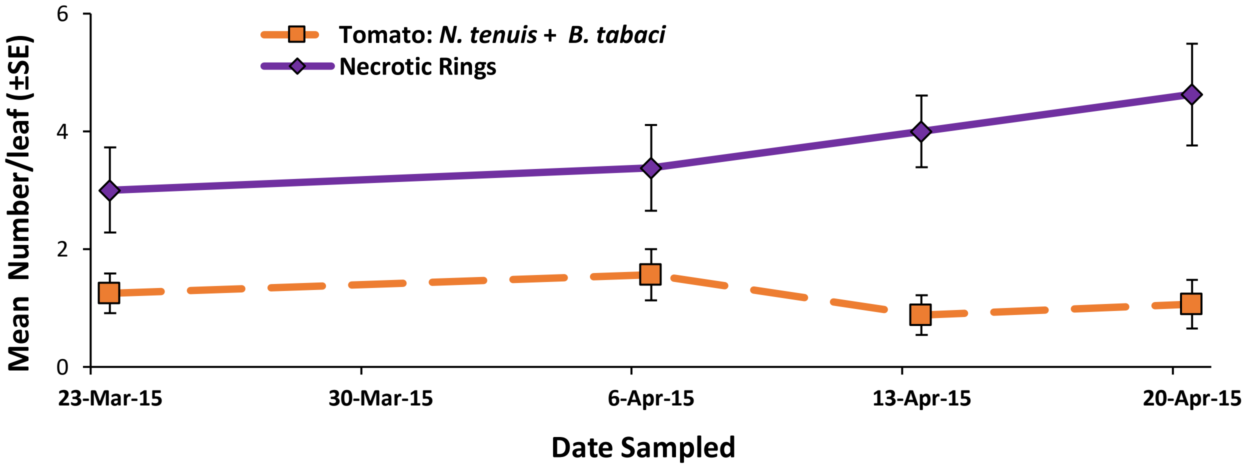

Supplement: Supplementary file 1 [file plants-11-02779-s001.zip › plants-1934246-supplementary.pdf]
